# Evaluation of complementary diagnostic tools for bovine tuberculosis detection in dairy herds from India

**DOI:** 10.14202/vetworld.2016.862-868

**Published:** 2016-08-16

**Authors:** Mukesh Kumar Thakur, Dharmender Kumar Sinha, Bhoj Raj Singh

**Affiliations:** 1 School of Public Health & Zoonoses, Guru Angad Dev Veterinary & Animal Sciences University, Ludhiana, Punjab, India; 2 Division of Epidemiology, Indian Veterinary Research Institute, Izatnagar, Bareilly, Uttar Pradesh, India

**Keywords:** bovine tuberculosis, culture isolation, *Mycobacterium tuberculosis* complex, polymerase chain reaction, single intradermal tuberculin testing

## Abstract

**Aim:**

A cross-sectional study was undertaken to know the herd prevalence and evaluate the single intradermal tuberculin testing (SITT), culture isolation, and polymerase chain reaction (PCR) analysis for the diagnosis of bovine tuberculosis (TB).

**Materials and Methods:**

A total of 541 cows of three dairy farms of Bareilly and Mukteshwar were screened by SITT followed by collection of pre-scapular lymph node (PSLN) aspirates (71), milk (54), and blood (71) samples from reactor animals. These clinical samples were processed for culture isolation and direct PCR-based identification and species differentiation.

**Results:**

Out of 541 cows screened by SITT, 71 (13.12%) animals were found positive. Mycobacteria were isolated from 3 (4.22%) PSLN aspirate but not from any cultured milk and blood samples. 28 (39.43%) PSLN aspirate and 5 (9.25%) milk samples were positive for *Mycobacterium* TB (MTB) complex (MTC) by PCR amplification for the IS6110 insertion sequence; however, blood samples were found negative. For species differentiation, multiplex-PCR using 12.7 kb primers was conducted. Out of 28 PSLN aspirate, *Mycobacterium bovis* was detected in 18 (64.28%) and MTB in 8 (28.57%), whereas 2 aspirate samples (7.14%) were positive for both the species. All the five milk positive samples were positive for *M. bovis*.

**Conclusion:**

Direct detection of bovine TB by a molecular-based method in dairy animals after preliminary screening was appeared to be more sensitive and specific compared to the conventional method (i.e., culture isolation). Its application in form of serial testing methodology for the routine diagnosis and thereafter, culling of infected stock may be suggested for the control programs in dairy herds. The PSLN aspirate was found to be the most suitable specimen for culture isolation and PCR-based detection of *Mycobacterium* spp. among live infected animals.

## Introduction

Bovine tuberculosis (TB) is an infectious chronic disease of cattle caused by *Mycobacterium bovis*, the organism of major concern worldwide because of its high economic impact on livestock industry due to mortality, decreased production, carcass condemnation, and zoonotic potential. *M. bovis* is one of the members of *Mycobacterium* TB (MTB) complex (MTC) that also includes MTB, *Mycobacterium microti*, *Mycobacterium africanum*, *Mycobacterium cannetti* [1], *Mycobacterium pinnipedi* [2], and *Mycobacterium caprae* [3]. According to the World Health Organization, bovine TB is a neglected zoonosis in many countries leading to a significant load of human TB due to *M. bovis*. In developed countries, mandatory pasteurization of milk along with stringent test and slaughter policy has resulted in the dramatic decline of *M. bovis* associated human TB [4]. In developing countries, however, animal TB is widely distributed, control measures are not applied or are applied sporadically, and pasteurization is rarely practiced [5]. Therefore, there is a need to reorient the available diagnostic tools, approaches, and policies on animal husbandry to achieve the disease-free or at least low incidence status in India.

The diagnosis of bovine TB depends on clinical manifestations, incidental necropsy evidence, tuberculin skin testing, and culture isolation methods, etc. In addition, lymphocyte proliferations, gamma interferon assay, and indirect enzyme-linked immunosorbent assay [6] are also available. The molecular diagnostic method *viz*. polymerase chain reaction (PCR), which can be used on live animal’s lymph node aspirate [7], milk [8], blood [9], and nasal secretions [10], is rapid, accurate, and quite promising. There is currently no single test which will fulfill all the criteria necessary to identify all infected animals. Of necessity, a combination of approaches is likely to be needed to achieve an adequate level of diagnosis [11].

The disease control is aimed at detection and removal of infected animals to prevent the spread within and between farms [12]. The incidence of bovine TB has been observed to be rising, especially in Asian and African continents where standard of living is poor [13]. With this view, the objectives of the present study were to find the prevalence on herd basis and evaluate the available diagnostic tests for diagnosing the live infected animals.

## Materials and Methods

### Ethical approval

The present study was conducted after the approval of Institutional Animal Ethics Committee, IVRI, Bareilly.

### Study area and population

**Figure-1:**
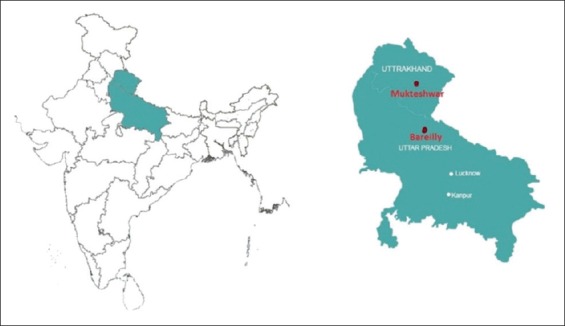
Study area – Bareilly (Uttar Pradesh) and Mukteshwar (Uttarakhand) marked by red dots.

The present study was conducted in three dairy farms (541 animals) of two locations, *viz*., Bareilly and Mukteshwar (Figure-1). In Bareilly, the study was conducted at one organized farm with 407 Jersey and Holstein Friesian crossbreds and another one at Gaushala with 35 non-descript cows, whereas at Mukteshwar organized dairy farm had 99 Jersey crossbred cows. There was regular annual single intradermal tuberculin testing (SITT) conducted at Mukteshwar farm while at Bareilly dairy farm SITT conducted occasionally that too without any follow-up actions, whereas at Gaushala SITT never conducted.

### Screening of dairy herds

The preliminary screening with SITT was done using purified protein derivative (PPD) (Division of Biological Products, IVRI, Izatnagar) using standard protocol [14]. Briefly, 2 inch^2^ × 2 inch^2^ area on the left side of neck was shaved and skin thickness measured with Vernier caliper. The 0.1 ml/animal (2000 IU) of bovine PPD (1 mg/ml) was injected via intradermal route. The tuberculin reaction was observed after 72 h, and results were interpreted based on skinfold thickness; more than 4 mm with or without clinical signs (positive reactor), no or <2 mm (negative reactor), between 2 and 4 mm with or without clinical signs (doubtful).

### Culture isolation

The samples, *viz*., pre-scapular lymph node (PSLN) aspirate (71) in normal saline, milk (54) and blood (71) in ethylenediaminetetraacetic acid (EDTA) from positive reactors (71) were collected aseptically and brought to laboratory on ice and stored at −20°C until further processing. The half portion of samples was used for the culture isolation and the rest for direct PCR analysis. About 30 ml milk taken as sample, which was centrifuged at 3000 rpm for 30 min at 4°C. The milk sediment along with the top cream layer was used for isolation, whereas whey portion was discarded. The decontamination of samples was carried out by mixing equal volumes of PSLN aspirate (0.5 ml), milk concentrate (2 ml), and blood (5.0 ml) with 4% NaOH solution as per modified Petroff’s method [15]. About 100 µl inoculum was seeded on Lowenstein-Jensen media (HiMedia) supplemented with pyruvate (0.5%) and glycerol separately for enhancing the growth of *M. bovis* and MTB, respectively, and incubated at 37°C in slanted position for 8-12 weeks with weakly observation for the presence of growth. The growth smears were prepared and stained by Ziehl–Neelsen method to differentiate acid-fast *Mycobacterium* spp. from non-acid-fast contaminants [16].

### PCR confirmation of culture isolates

The DNA from culture isolates was extracted by a conventional method, *viz*., phenol-chloroform-isoamyl alcohol and stored in 50 µl of ×1 Tris-EDTA (TE) buffer at −20°C until use. Insertion sequence IS*6110* for genus identification of MTC with amplicon size 445 bp and 12.7 kb fragment primers based multiplex-PCR for the species differentiation with product sizes 389 bp (MTB), and 823 bp (*M. bovis*) were used. The primer sequences based on previously described sequences for IS*6110* [17] and 12.7 kb [18] were used. IS*6110* (F: 5’-GACCACGACCGAAGAATCCGCTG-3’ and R: 5’-CGGACAGGCCGAGTTTGGTCATC-3’); 12.7 kb (F: 5’- CACCCCGATGATCTTCTGTT-3’, R1: 5’-GCCAGTTTGCATTGCTATT-3’ and R2: 5’-GACCCGCTGATCAAAGGTAT-3’).

The reaction mixture (25 µl) for MTC detection consisted of 2.5 µl ×10 PCR buffer, 1.5 mM MgCl_2_, 200 µM deoxynucleotide triphosphate (dNTPs) mixture, 1 U Taq DNA polymerase (Bangalore Genei, India), 10 pmol IS*6110* primer (Eurofins, Bengaluru), and 5 µl DNA template. While for species differentiation, 2.5 µl ×10 PCR buffer, 2.0 mM MgCl_2_, 800 µM dNTPs mixture, 12.7 kb primers (15 pmol-F, 7.5 pmol-R1, and 7.5 pmol-R2) (Eurofins), 1 U Taq DNA polymerase, and 5 µl DNA template were used. The PCR conditions for IS*6110* primers were consisted of initial denaturation at 94°C for 5 min followed by 38 cycles of DNA amplification with denaturation, annealing, and extension at 94°C, 56°C, and 72°C, respectively, each for 30 s followed by final extension at 72°C for 10 min with product holding at 4°C. Similarly, for 12.7 kb primers, the initial denaturation was carried out at 94°C for 5 min followed by 35 cycles of DNA amplification with denaturation and annealing at 94°C and 59°C, respectively, each for 45 s with extension at 72°C for 1 min followed by final extension at 72°C for 10 min with final sample holding at 4°C. The amplified products were analyzed by electrophoresis in ×1 Tris-acetate EDTA for 1 h at 70 V. The PCR products, DNA marker, and controls were allowed to run in 1.5% agarose gel which thereafter visualized under ultraviolet light at 260 nm.

### Direct PCR-based detection from clinical samples

The DNA was extracted from the samples (PSLN aspirates - 71, milk - 54, and blood - 71) by phenol-chloroform-isoamyl alcohol method with some modifications.

#### DNA extraction from PSLN aspirates

The 500 µl PSLN aspirate was freeze-thawed for 3 cycles (heat in boiling water for 10 min followed by freezing at −20°C for 15 min). The lysozyme 40 µl (20 mg/ml) was added and incubated at 37°C for overnight. Thereafter, 80 µl of 10% sodium dodecyl sulfate and 5 µl of proteinase K (20 mg/ml) were added and incubated at 65°C for 2 h. Subsequently, 100 µl of 5 M sodium chloride (NaCl) and 85 µl of cetyl trimethyl ammonium bromide-NaCl (prewarmed to 65°C) were added and incubated at 65°C for 30 min. Equal volume of phenol: chloroform:isoamyl alcohol (25:24:1) was added and mixed well and centrifuged at 10,000 rpm for 10 min. The aqueous layer was transferred to fresh micro-centrifuge and repeated the step. To the aqueous layer, 0.8 V of isopropanol was added and DNA was allowed to precipitate at −20°C for overnight. The DNA was pelleted by centrifuging at 10,000 rpm for 10 min at 4°C, and supernatant was discarded. The DNA pellet was washed with 500 µl of 70% ethanol, mixed pellet thoroughly, and centrifuged at 10,000 rpm for 10 min at 4°C. Supernatant was discarded, and micro-centrifuge tubes were inverted on tissue paper to dry off. The dried pellet was re-suspended in 30 µl in TE buffer, and DNA was stored at −20°C.

#### DNA extraction from milk

Milk sample (2 ml) was centrifuged at 3000 rpm for 45 min and whey was discarded. Milk fat and sediment were mixed with 400 µl TE buffer followed by freeze-thawed for 3 cycles. The rests of further steps were the same as for the PSLN aspirates.

#### DNA extraction from blood

The DNA from blood samples was extracted according to the method described by Bates [19] with some modifications.

#### MTC genus and species differentiation

The amplification of DNA extracted from samples was carried out for the MTC genus identification and species differentiation according to the above-described PCR conditions.

### Comparison of tests results

The performance of culture isolation and PCR was evaluated by recording the relative sensitivity and specificity, predictive value, true and apparent prevalence, accuracy, likelihood ratio (LR), concordance, and odd ratio [20] with 2 × 2 contingency table for their validation.

## Results and Discussion

Bovine TB is endemic in India. The tuberculin skin testing with PPD is the OIE recommended test for screening against TB [14]. In India, there are number of studies conducted to know the level of bovine TB and realize the importance of control measures. For eradication, the testing of entire stock at quarterly intervals firstly, then at 6 monthly intervals and later, when incidence was low, at yearly interval with segregation of tuberculin reactors at separate locations were recommended [21]. However, in reality, these recommendations were not followed in true senses that led to the increased level of disease. Under these situations, there appears stark chance of TB elimination from Indian dairy populations. Mukherjee [22] highlighted that if a farm does not follow the recommendations of the OIE or the Bureau of Indian Standards for the control of TB, a high prevalence situation could be generated over a period hence insisted the need for change in breeding strategies and policies on farms.

The present study has focused the detection of infected animals using antemortem techniques. In SITT, 71 (13.12%) cows found tuberculin reactors from three dairy herds (541 animals) for bovine TB (Table-1), which was within the range of earlier reports from India [23-25]. The highest proportion of reactors was found in Bareilly organized dairy farm (16.46%) followed by Gaushala (11.42%). However, none of Mukteshwar dairy cattle were reactors. The tuberculin testing is being used extensively throughout the world but is compromised by variable sensitivity and specificity values. The mammalian tuberculin used is not sufficiently specific to differentiate between the reaction due to *M. bovis* and other members of the genus *Mycobacterium* including vaccination or *Nocardia* sp. There are reports of *M. avium* subsp. *paratuberculosis* prevalence among common livestock species in this region [26,27] which is known for causing cross-reactions in SITT and, in turn, increases the false positivity. The presence of no-visible-lesion reactors in tuberculin testing more than 10% also signifies the need of other tests [28]. Furthermore, reactivity is influenced by its inability to detect the cases of minimal sensitivity, aged animals, recently calved cattle, early infection state, anergic cases, etc. [28] including extrinsic factors (e.g., method used, PPD quality, and manual reading of hypersensitivity) associated with the method. Furthermore, it has been recently showed that the genetic background of the animal can influence the reaction to tuberculin [29]. Therefore, such limitations of skin test insist the presence of other compensatory methods.

**Table-1:** List of dairy farms screened through SITT

Dairy farms	Animals tested	SITT positive (%)
Organized dairy farm - Bareilly	407	67 (16.46)
Gaushala - Bareilly	35	4 (11.42)
Organized dairy farm - Mukteshwar	99	0 (0.00)
Total	541	71 (13.12)

SITT=Single intradermal tuberculin testing

As there was no history of regular herd screening and other measures in Bareilly organized farm, which might be the possible underlying causes for the higher prevalence, whereas the Gaushala maintaining only a few productive and majority of non-breeding discarded stock, might explain that these animals incurred infection either from their previous respective locations or diseased animals living in contact at Gaushala. In Mukteshwar farm, the absence of reactors might be due to its regular herd screening practice, isolated location, internal replacement of animals, etc.

**Figure-2:**
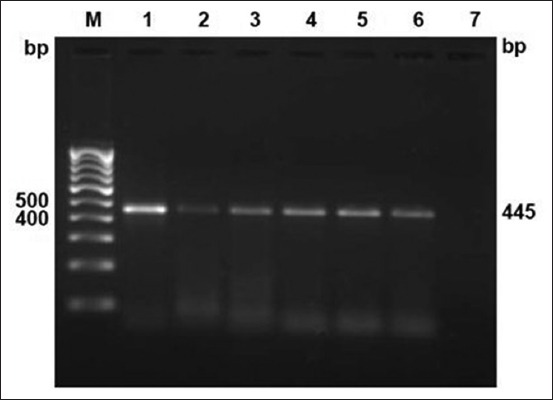
Culture isolates confirmation by polymerase chain reaction targeting IS*6110* sequence. Lane M: 100 bp DNA ladder; positive control (Lane 1: Mycobacterium *tuberculosis* H37Rv; Lane 2: *Mycobacterium bovis* BCG); Lane 3-6: Culture isolates (duplicates).

**Figure-3:**
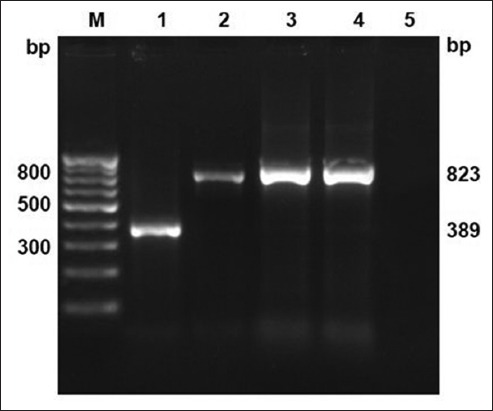
Culture isolates speciation by multiplex-polymerase chain reaction targeting 12.7 bp fragment sequence. Lane M: 100 bp DNA ladder; positive control (Lane 1: *Mycobacterium tuberculosis* H37Rv; Lane 2: *Mycobacterium bovis* BCG); Lane 3-4: Culture isolates *M. bovis* at 823 bp.

The culture isolation was carried out on PSLN aspirates (71), milk (54), and blood samples (71) collected from reactor animals. Three *Mycobacterium* spp. (4.22%) could be isolated from PSLN aspirates collected from Bareilly organized dairy farm; however, none of the milk and blood samples found culture positive. On PCR analysis, two isolates were positive for MTC (Figure-2) and one for non-TB *Mycobacterium* species. Further, on species differentiation by multiplex-PCR, both isolates were positive for *M. bovis* (Figure-3). Likewise, Ali *et al*. [30] examined 105 milk samples but could not find either of samples positive. Similarly, low isolation rates from milk were also recorded by Ofukwu *et al*. [31] and Hassanain *et al*. [32]. Unlike the present study, comparatively higher isolation rate from the aspirate sample was reported in India [15]. However, the aspirate sample in the present study was qualified better among the constraints of availability of suitable samples for isolation and PCR-based diagnosis. TB in cattle is principally a pulmonary disease; therefore, Grange and Yates [33] have shown that only 1% tuberculous cows excrete tubercle bacilli in milk. Similarly, from the present study findings, it may be suggested that the bacillary discharge in milk is intermittent in nature; hence, continuous screening in suspected cases is required before their final disposal.

The isolation from blood was also attempted in this study, but without success, which may suggest blood as unsuitable sample for isolation in bovines. Although, the isolation of *Mycobacterium* sp. is specific, reliability still questionable as success varies with bacillary load, intermittent shedding pattern, decontamination process followed, co-presence of rapid growers, long incubation period, etc. [34]. These might be the possible reasons for lower isolation rates recorded in the present study.

**Figure-4:**
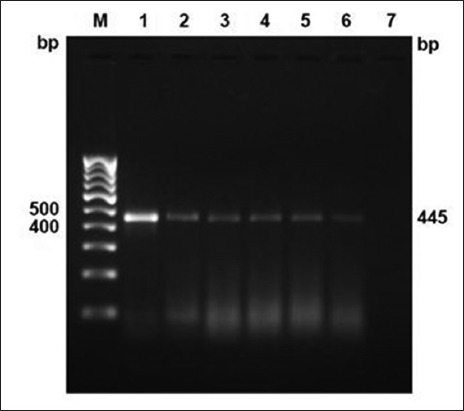
Direct polymerase chain reaction detection of *Mycobacterium* sp. in clinical samples (pre-scapular lymph node aspirates and milk) targeting IS*6110* sequence for members of *Mycobacterium* TB (MTB) complex at 445 bp. Lane M: 100 bp DNA ladder; positive control (Lane 1: MTB H37Rv; Lane 2: *Mycobacterium bovis* BCG); Lane 3-6: MTC in clinical samples.

**Figure-5:**
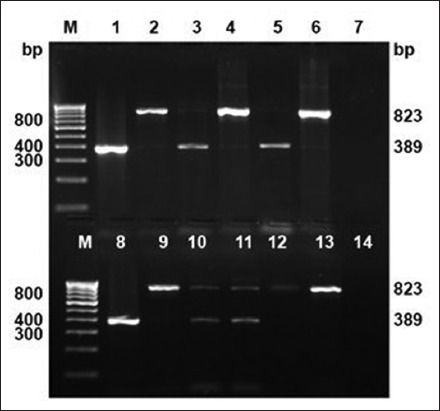
Species differentiation of *Mycobacterium* TB (MTB) complex members in clinical samples (pre-scapular lymph node aspirates and milk) by multiplex-polymerase chain reaction targeting 12.7 kb fragment sequence (MTB - 389 bp, *Mycobacterium bovis* - 823 bp). Lane M: 100 bp DNA ladder; positive control (Lane 1: MTB H37Rv; Lane 2: *M. bovis* BCG); Lane 3, 5, and 8: MTB (389 bp); Lane 4, 6, 9, 12, and 13: *M. bovis* (823 bp); Lane 10 and 11: Mixed (MTB and *M. bovis*).

Around 28 (39.43%) PSLN aspirate and 5 (9.25%) milk samples were positive for MTC by PCR amplification for the IS*6110* insertion sequence; however, blood samples were found negative (Table-2 and Figure-4). Out of total five milk positive samples, four were among the 28 PSLN aspirate positive animals, whereas one was among aspirate negative samples. Hence, total numbers of positive animals were 29 out of 71 tuberculin reactors. The two culture isolates of *M. bovis* were also from 28 animals showing positive PSLN aspirate samples in direct PCR detection. Among 71 SITT reactors, 26.76% (19/71) *M. bovis* and 11.26% (8/71) MTB-infected animals were found by 12.7 kb multiplex-PCR with 2.81% (2/71) co-infected with both the species (Table-2 and Figure-5). The share of MTB was 34.48% (10/29) among MTC positive samples.

**Table-2:** Direct PCR-based analysis of clinical samples from tuberculin reactors.

Clinical samples	Positive samples based on direct PCR analysis				Total (%)

	IS*6110*	12.7 kb multiplexPCR			
	
	MTC	*M. bovis*	MTB	Both	
PSLN aspirate (71)	28	18	8	2	28/71 (39.43)
Milk (54)	5	5	-	-	5/54 (9.25)
Blood (71)	-	-	-	-	0/71 (0.00)

MTC=*Mycobacterium tuberculosis* complex, MTB=*Mycobacterium tuberculosis*, *M. bovis*=*Mycobacterium bovis*, PCR=Polymerase chain reaction

In the present study, PSLN aspirate was found to be a relatively superior sample for isolation and/or for direct PCR-based detection, which is also being corroborated by the observation of Srivastava *et al*. [15]. Whereas using milk for isolation and/or direct PCR detection, we need to develop strict and robust laboratory protocols due to variable bacillary discharge in milk as was revealed by lower detection rates from milk in the present study. Similarly, for the blood, we cannot rely on its suitability in bovines though it was proven satisfactory specimen in human.

The evaluation of PCR was done by taking culture isolation and SITT as reference tests and compared with them separately (Table-3). With SITT, the PCR has recorded higher relative specificity (100%) and positive predictive values (100%) than comparing with culture isolation where 60.29% and 6.89% were the respective values. Whereas, the relative sensitivity (40.84%) and negative predictive values (91.79%) were comparatively lower for PCR comparing with SITT. Similarly, other parameters, *viz*., prevalence, accuracy, LR, and concordance were also recorded and proved significant in PCR-SITT combination. The accuracy of PCR-SITT combination (92.23%) was also recorded better than the PCR-culture combination. The LR^+^ was infinite (highest) for PCR when comparing with SITT than comparing with culture isolation (1.67), and LR^−^ was 0.592 when comparing with SITT, which depicts its perfection as a diagnostic test when applied along with SITT. The concordance between PCR and SITT was also found higher (0.922) which signifies their usage in serial testing methodology. The calculated odd ratio of PCR in comparison to SITT was also found maximum (infinity), indicating its excellent discrimination power. Therefore, screening the herd initially by SITT followed by PCR-based diagnosis using PSLN aspirate sample of the positive reactors would increase the specificity in the diagnosis of bovine TB. Furthermore, it is also useful in species identification to realize the zoonotic risk of animal infections.

**Table-3:** Comparison of PCR with culture and SITT.

PCR/culture					PCR/SITT				
	
		Culture					SITT		
	
		+	-				+	-	
PCR	+	2	27	29	PCR	+	29	0	29
	-	1	41	42		-	42	470	512
		3	68	71			71	470	541

PCR=Polymerase chain reaction, SITT=Single intradermal tuberculin testing

The ultimate problem in India lies with the disposal of diseased/infected cases once diagnosed, where due to socio-economic and religious constraints, the cow slaughter is not allowed. As number of the European countries has achieved the TB-free status due to their stringent test and slaughter eradication policy; therefore, our animal health policy makers should also have to focus on this area in their priority issues. The compensation from the government should be provided to farmers for the maintenance of such potentially infected animals in specially designated herd locations up to their natural life span so that the source of infection to rest of herd mates and human beings can be minimized.

## Conclusion

The result of present study signifies the suitability of SITT-PCR combination for the diagnosis of bovine TB. These methods may be utilized on serial test methodology basis for achieving higher specificity in test results as needed for the control of the disease. The PSLN aspirate was found appropriate specimen for culture isolation and direct PCR-based detection from live infected animals.

## Authors’ Contributions

MKT and DKS designed the research problem in consultancy of BRS. MKT and DKS carried out SITT and other lab works. MKT prepared the manuscript. Finalization of the manuscript was done by DKS and BRS. All authors read and approved the final version.

## References

[1] Van Soolingen D, Hoogenboezem T, de Haas P.E.W, Hermans P.W.M, Koedam M.A, Teppema K.S, Brennan P.J, Besra G.S, Portaels F, Top J, Schouls L.M, Van Embden J.D.A (1997). A novel pathogenic taxon of the *Mycobacterium tuberculosis* complex, Canetti: Characterization of an exceptional isolate from Africa. Int. J. Syst. Bacteriol.

[2] Cousins D.V, Bastida R, Cataldi A, Quse V, Redrobe S, Dow S, Duignan P, Murray A, Dupont C, Ahmed A, Collins D.M, Butler W.R, Dawson D, Rodriguez D, Loureiro J, Romano M.I, Alito A, Zumarraga M, Bernardelli A (2003). Tuberculosis in seals caused by a novel member of the *Mycobacterium tuberculosis* complex *Mycobacterium pinnipedii* sp. nov. Int. J. Syst. Evol. Microbiol.

[3] Aranaz A, Cousins D, Mateos A, Dominiguez L (2003). Elevation of *Mycobacterium tuberculosis* subsp *Caprae* Aranaz *et al*. 1999 to species rank as *Mycobacterium caprae* comb. nov., sp. nov. Int. J. Syst. Evol. Microbiol.

[4] Palmer M.V, Thacker T.C, Waters W.R, Gort C.A, Corner L.A (2012). *Mycobacterium bovis*: A model pathogen at the interface of livestock, wildlife, and humans. Vet Med Int.

[5] Cosivi O, Grange J.M, Daborn C.J, Raviglione M.C, Fujikura T, Cousins D, Robinson R.A, Huchzermeyer F.A.K, de Kantor I, Meslin F.X (1998). Zoonotic tuberculosis due to *Mycobacterium bovis* in developing countries. Emerg Infect Dis.

[6] Thakur M.K, Sinha D.K, Singh B.R (2015). Evaluation of PPD based ELISA in the diagnosis of bovine tuberculosis. J Anim Res.

[7] Mishra A, Singhal A, Chauhan D.S, Katoch V.M, Srivastava K, Thakral S.S, Bharadwaj S.S, Sreenivas V, Prasad H.K (2005). Direct detection and identification of *Mycobacterium tuberculosis* and *Mycobacterium bovis* in bovine samples by a novel nested PCR assay: Correlation with conventional techniques. J Clin Microbiol.

[8] Lermo A, Liébana S, Campoy S, Fabiano S, García M.I, Soutullo A, Zumárraga M.J, Alegret S, Pividori M.I (2010). A novel strategy for screening - Out raw milk contaminated with *Mycobacterium bovis* on dairy farms by double-tagging PCR and electrochemical genosensing. Int. Microbiol.

[9] Dubey S (2015). Molecular Epidemiology of Chronic Bacterial Diseases in Mithun M.V.Sc. Thesis.

[10] Figueiredo E.E.S, Carvalho R.C.T, Silvestre F.G, Lilenbaum W, Fonseca L.S, Silva J.T, Paschoalin V.M.F (2010). Detection of *Mycobacterium bovis* DNA in nasal swabs from tuberculous cattle by a multiplex PCR. Braz J Microbiol.

[11] Medeiros L.S, Marassi C.D, Figueiredo E.E.S, Lilenbaum W (2010). Potential application of new diagnostic methods for controlling of bovine tuberculosis in Brazil. Braz J Microbiol.

[12] Morrison W.I, Bourne F.J, Cox D.R, Donnelly C.A, Gettinby G, McInerney J.P, Woodroffe R (2000). Pathogenesis and diagnosis of infections with Mycobacterium bovis in cattle. Vet. Rec.

[13] Ameni G, Bonnet P, Tibbo M (2003). A cross-sectional study of bovine tuberculosis in selected dairy farms in Ethiopia. Int. J. Appl. Res. Vet. Med.

[14] Office Internationale des Epizooties (2009). Manual of Diagnostic Tests and Vaccines for Terrestrial Animals.

[15] Srivastava K, Chauhan D.S, Gupta P, Singh H.B, Sharma V.D, Yadav V.S, Sreekumaran Thakral S.S, Dharamdheeran J.S, Nigam P, Prasad H.K, Katoch V.M (2008). Isolation of *Mycobacterium bovis* and *M. Tuberculosis* from cattle of some farms in North India - Possible relevance in human health. Indian J. Med. Res.

[16] Grange J.M (1988). The genus mycobacteria and species of Mycobacteria. In: Mycobacteria and Human Disease.

[17] Thierry D, Cave M.D, Eisenach K.D, Crawford J.T, Bates J.H, Gicquel B, Guesdon J.L (1990). IS*6110* an IS - Like element of *Mycobacterium tuberculosis* complex. Nucleic Acids Res.

[18] Zumarraga M, Bigi F, Alito A, Romano M.I, Cataldi A (1999). A 12.7 kb fragment of the *Mycobacterium tuberculosis* genome is not present in *Mycobacterium bovis*. Microbiology.

[19] Bates I (1974). Isolation and purification of deoxyribonucleic acid from *Mycobacteria*. Acta Pathol. Microbiol. Scand. B.

[20] Thrusfield M (2005). Diagnostic testing. In: Veterinary Epidemiology.

[21] Chauhan H.V.S, Dwivedi P.D, Chauhan S.S, Kalra D.S (1974). Tuberculosis in animals in India - A review. Indian J. Tuberc.

[22] Mukherjee F (2006). Comparative prevalence of tuberculosis in two dairy herds in India. Rev. Sci. Tech. Off. Int. Epiz.

[23] Thakur A, Sharma M, Katoch V.C, Dhar P, Katoch R.C (2010). A study on the prevalence of bovine tuberculosis in farmed dairy cattle in Himachal Pradesh. Vet. World.

[24] Ganesan P.I (2012). Comparative efficacy of single intradermal test and gamma interferon assay in the diagnosis of bovine tuberculosis. Indian Vet. J.

[25] Filia G, Leishangthem G.D, Mahajan V, Singh A (2016). Detection of *Mycobacterium tuberculosis* and *Mycobacterium bovis* in Sahiwal cattle from an organized farm using ante mortem techniques. Vet. World.

[26] Sonawane G.G, Narnaware S.D, Tripathi B.N (2016). Molecular epidemiology of *Mycobacterium avium* subspecies *paratuberculosis* in ruminants in different parts of India. Int J Mycobacteriol.

[27] Kaur P, Filiaa G, Singh S.V, Patil P.K, Ravi Kumar G.V.P, Sandhu K.S (2011). Molecular epidemiology of *Mycobacterium avium* subspecies paratuberculosis: IS900 PCR identification and IS1311 polymorphism analysis from ruminants in the Punjab region of India. Comp. Immunol. Microbiol. Infect. Dis.

[28] Radostits O.M, Gay C.C, Hinchcliff K.W, Constable P.D (2007). Veterinary Medicine: A Text Book of the Diseases of Cattle, Horses, Sheep, Pigs and Goats.

[29] Amos W, Brooks-Pollock E, Blackwell R, Driscoll E, Nelson-Flower M, Conlan A.J (2013). Genetic predisposition to pass the standard SICCT test for bovine tuberculosis in British cattle. PLoS One.

[30] Ali S, Khan I.A, Mian M.S, Rana W (2005). Detection of Mycobacteria from milk of cattle and buffaloes at government livestock farms. Pak. J. Agric. Sci.

[31] Ofukwu R.A, Oboegbulem S.I, Akwuobu C.A (2008). Zoonotic *Mycobacterium species* in fresh cow milk and fresh skimmed, unpasteurised market milk (nono) in Makurdi, Nigeria: Implications for public health. J. Anim. Plant Sci.

[32] Hassanain N.A, Hassanain M.A, Soliman Y.A, Ghazy A.A, Ghazyi Y.A (2009). Bovine tuberculosis in a dairy cattle farm as a threat to public health. Afr. J. Microbiol. Res.

[33] Grange J.M, Yates M.D (1994). Zoonotic aspects of *Mycobacterium bovis* infection. Vet. Microbiol.

[34] Kidane D, Olobo J.O, Habte A, Negesse Y, Aseffa A, Abate G, Yassin M.A, Bereda K, Harboe M (2002). Identification of the causative organism of tuberculous lymphadenitis in Ethiopia by PCR. J Clin Microbiol.

